# CATCH: a randomised clinical trial comparing long-term tinzaparin versus warfarin for treatment of acute venous thromboembolism in cancer patients

**DOI:** 10.1186/1471-2407-13-284

**Published:** 2013-06-13

**Authors:** Agnes YY Lee, Rupert Bauersachs, Mette S Janas, Mikala F Jarner, Pieter W Kamphuisen, Guy Meyer, Alok A Khorana

**Affiliations:** 1 University of British Columbia and Vancouver Coastal Health, 2775 Laurel Street, 10th floor, Vancouver, BC V5Z 1M9, Canada; 2 Max-Ratschow-Klinik für Angiologie, Klinikum Darmstadt GmbH, Darmstadt, Germany; 3 LEO Pharma, Ballerup, Denmark; 4 University of Groningen, Groningen, Netherlands; 5 Hôpital européen Georges-Pompidou, Paris, France; 6 Taussig Cancer Institute, Cleveland Clinic, Cleveland, OH, USA

**Keywords:** Venous thromboembolism, Cancer, LMWH, Tinzaparin, Warfarin, CATCH, Recurrent, Symptomatic, Incidental, Health-related quality of life

## Abstract

**Background:**

Low-molecular-weight heparin (LMWH) is recommended and commonly used for extended treatment of cancer-associated thrombosis (CAT), but its superiority over warfarin has been demonstrated in only one randomised study. We report here the rationale, design and *a priori* analysis plans of Comparison of Acute Treatments in Cancer Haemostasis (CATCH; NCT01130025), a multinational, Phase III, open-label, randomised controlled trial comparing tinzaparin with warfarin for extended treatment of CAT.

**Methods/Design:**

The primary objective is to assess the efficacy of tinzaparin in preventing recurrent venous thromboembolism (VTE) in patients with active cancer and acute, symptomatic proximal deep vein thrombosis and/or pulmonary embolism. The secondary objectives are to determine: safety of tinzaparin given over 6 months; clinical and laboratory markers for recurrent VTE and/or major bleeding; 6-month overall mortality; incidence and severity of post-thrombotic syndrome; patient-reported quality of life; and healthcare resource utilisation. Nine hundred patients are randomised to receive tinzaparin 175 IU/kg once daily for 6 months or initial tinzaparin 175 IU/kg once daily for 5–10 days and dose-adjusted warfarin (target INR 2.0–3.0) for 6 months. The primary composite outcome is time to recurrent VTE, including incidental VTE and fatal pulmonary embolism. All patients are followed up to 6 months or death, whichever comes sooner. Blinded adjudication will be performed for all reported VTE, bleeding events and causes of death. Efficacy will be analysed using centrally adjudicated results of all patients according to intention-to-treat analysis. An independent Data Safety Monitoring Board is reviewing data at regular intervals and an interim analysis is planned after 450 patients have completed the study.

**Discussion:**

The results will add significantly to the knowledge of the efficacy, safety and cost effectiveness of tinzaparin in the prevention of recurrent VTE in patients with cancer and thrombosis. Prospective data will emerge on the clinical significance of incidental VTE and risk stratification in patients with CAT. Results on post-thrombotic syndrome, quality of life and healthcare resource utilisation will inform decision makers on how to secure better patient care. If tinzaparin is shown to be more effective than warfarin, CATCH will provide valuable confirmatory data to support the use of the LMWH tinzaparin for extended treatment of CAT.

## Background

### Cancer and venous thromboembolism

Cancer patients are known to be at higher risk of developing venous thromboembolism (VTE) compared with the general population [1-3]. VTE is a major cause of morbidity and mortality and is the second most common cause of death in patients with cancer [4,5]. Moreover, with the increasing incidence and prevalence of cancer, combined with more aggressive, often thrombogenic treatment regimens and surgery, the prevalence of cancer-associated thrombosis can be expected to increase.

The incidence of symptomatic VTE in cancer patients varies widely, with reported rates of 2% to 30%. This variation is likely to reflect not only the natural history of different tumour types [6-8] but also the influence of tumour-related factors (e.g. tumour location, presence of distant metastases) [1,7,9,10], treatment-related factors (e.g. surgery, chemotherapy) [11,12] and patient-related factors (e.g. older age, co-morbidities, multiple surgeries) [9]. The true rate of VTE is probably even higher than reported rates as many cases remain undetected. As part of an increasing focus on staging procedures, treatment modalities, treatment outcomes and improving cancer survival, the significance of asymptomatic or incidental events is becoming increasingly recognised [13]. Indeed, during the past decade, computed tomography (CT) scans have been used with greater frequency for the routine assessment of cancer staging and disease monitoring and, concordant with this, there has been increased reporting of incidental VTE, especially pulmonary embolism (PE), in cancer patients [14]. Neither the prognostic impact nor the treatment of incidental PEs in cancer patients is completely understood, but the consensus is that these events should be treated [15]. This is supported by data showing that the prevalence of incidental PE is clinically relevant and potentially associated with an unfavourable outcome [16,17].

### Treatment of VTE in cancer patients

Guidelines in Europe and North America recommend long-term treatment of symptomatic VTE in all cancer patients [18,19]. Major treatment objectives are to diminish the acute symptoms of deep vein thrombosis (DVT) and/or PE, reduce recurrent thrombosis, and reduce both fatal and non-fatal PE. Treatment of VTE should also reduce the incidence of long-term sequelae, such as post-thrombotic syndrome (PTS). With improved oncology treatment options leading to longer life expectancy, the benefits of long-term treatment of VTE are becoming increasingly relevant to patients with cancer. This trend towards better care might be expected to have a positive impact on a patient’s quality of life (QoL); however, relatively little is known about this important topic and further investigation of patient-reported outcomes is warranted.

For the general population, the standard treatment for acute VTE consists of initial therapy with a low-molecular-weight heparin (LMWH) followed by longer-term treatment (3–6 months) with an oral vitamin K antagonist (VKA). Although this approach can be effective for many patients, cancer patients have a substantial risk of recurrent VTE. Several studies have reported incidences of recurrent VTE as high as 20% in patients with cancer [20,21]. However, studies on how to treat and identify those at risk of recurrent VTE are limited [22,23]. Moreover, the frequent monitoring and dose adjustments required for VKA treatment have a negative impact on QoL [24]. Oral anticoagulant therapy with VKAs or novel oral anticoagulants, e.g. dabigatran and rivaroxaban, may also be problematic in patients with cancer because of drug interactions, malnutrition, vomiting and mucositis, and renal and liver dysfunction, all of which can lead to unpredictable bioavailability and variable levels of anticoagulation. Patients with advanced cancer may also have problems swallowing oral medications. Furthermore, the efficacy and safety of novel oral anticoagulants have yet to be determined in cancer patients and there is no antidote to reverse the anticoagulant effect of these drugs in cases of severe bleeding or when acute surgical intervention is necessary.

Unlike VKAs, LMWHs have predictable pharmacokinetic profiles and very few drug interactions [25]. LMWHs have been shown to have clinical benefit over VKAs in the secondary prevention of VTE in cancer patients [26-29]. Consequently, major consensus evidence-based guidelines on anticoagulation recommend that LMWHs be used for 3–6 months for the treatment of acute VTE in patients with active cancer (ASCO [30], ESMO [31], NCCN [32] and ACCP [15]). Although only the CLOT study has demonstrated statistically significant reduction in symptomatic VTE using dalteparin compared with VKA [28] (Table 1), all three major LMWHs (dalteparin, enoxaparin and tinzaparin) are recommended in guidelines. In the CLOT trial, dalteparin was given at a full, therapeutic dose for the first month followed by 75–80% of the full dose from month 2 to 6. In contrast, full doses of LMWH were given throughout the entire treatment period in the CANTHANOX, ONCENOX and LITE studies (Table 1). Whether this latter approach would have resulted in lower risks of VTE recurrence or higher risks of bleeding in CLOT is unknown. None of these previous trials addressed questions regarding PTS, QoL, predictors of recurrence, and the cost effectiveness of LMWH monotherapy, all of which are important considerations for long-term therapy in a complex patient population with reduced life expectancy. Finally, the open-label design of published trials and the perceived patient preference of oral therapy over subcutaneous injections led the ACCP to downgrade its previous 1A recommendation from the 2008 guidelines to 2B in its most recent review [15]. In addition, the lack of confirmatory studies may explain the continued widespread use of VKAs [33,34]. To narrow these gaps in knowledge, and to provide additional data to support multiple guidelines that recommend prolonged treatment with LMWH as the preferred anticoagulant for cancer-associated thrombosis, we are conducting CATCH (Comparison of Acute Treatments in Cancer Haemostasis), a multinational, open-label, randomised controlled trial designed to compare tinzaparin (innohep®, LEO Pharma A/S) with warfarin for the extended treatment of cancer-associated VTE.

**Table 1:** **Randomised clinical trials of VTE treatment in patients with cancer (adapted from Khorana 2009**[18]**)**

**Study**	**n**	**Population**	**Treatments**	**Follow-up**	**Outcomes**	**Key limitations compared with the CATCH study design**
CLOT [28]	672	Acute symptomatic proximal DVT and/or PE	Dalteparin qd (5–7 days) + warfarin* (6 months)	6 months	**Recurrent VTE (primary)** 15.8% (W), 8.0% (D); *P* = 0.002	Full-dose dalteparin not maintained for entire 6-month treatment period
			Dalteparin qd (6 months)^†^		**Major bleeding** 4% (W), 6.0% (D); *P* = 0.27	No outcomes for PTS, HRQoL, predictors of recurrence, and healthcare resource utilisation
Main-LITE^‡^[27]	200	Proximal DVT	UFH + warfarin (6 days) then warfarin (3 months)	3 months and 12 months	**Recurrent VTE (primary)** 3 months: 10.0% (W), 6.0% (T) 12 months: 16.0% (W), 7.0% (T); *P* = 0.044	Duration of randomised treatment only 3 months
			Tinzaparin qd (3 months)		**Major bleeding** 3 months: 7.0% (W), 7.0% (T)	Modest sample size with limited statistical power
CANTHANOX [29]	146	DVT and/or PE	Enoxaparin qd (initial) + warfarin (3 months)	3 months	**Treatment failure**^**§ **^**(primary)** 21.1% (W), 10.5% (E); *P* = 0.09	Composite primary endpoint (recurrent VTE and major bleeding)
			Enoxaparin qd (3 months)		**Major bleeding** 16.0% (W), 7.0% (E); *P* = 0.09	Duration of randomised treatment only 3 months Small study with limited statistical power Trial stopped early because of slow recruitment
ONCENOX [26]	122	Acute symptomatic VTE	Enoxaparin LD bid (5 days) + warfarin (6 months)	6 months	**Recurrent VTE (secondary)** 10.0% (W), 6.9% (LD), 6.3% (HD)	Recurrent VTE was only a secondary objective (study did not meet its primary objective, which was to recruit the necessary number of patients within a 12-month time frame) Small study with limited statistical power
			Enoxaparin LD bid (5 days) then LD qd (6 months)			
			Enoxaparin LD bid (5 days) then HD qd (6 months)		**Major bleeding** 2.9% (W), 6.5% (LD), 11.1% (HD)	

*Except in Spain and The Netherlands, where acenocoumarol was used; ^†^full dose (200 IU/kg) for first month then reduced dose (~150 IU/kg) for the remaining 5 months; ^‡^cancer subpopulation; ^§^composite endpoint of recurrent VTE and/or major bleeding within 3-month treatment period.

Abbreviations: *bid* twice daily; *D* dalteparin, *DVT* deep vein thrombosis, *E* enoxaparin, *HD* high dose (1.5 mg/kg), *HRQoL* health-related quality of life, *LD* low dose (1.0 mg/kg), *PE* pulmonary embolism, *PTS* post-thrombotic syndrome, *qd* each day, *T* tinzaparin, *UFH* unfractionated heparin, *VTE* venous thromboembolism, *W* warfarin.

### Main study objectives

The main aim of CATCH is to compare the efficacy of tinzaparin with warfarin in preventing the recurrence of VTE in patients with active cancer. Secondary objectives are: to assess the safety of long-term tinzaparin; to identify clinical risk factors for recurrent VTE and bleeding; to assess overall mortality at 6 months; to identify the possible role of coagulation parameters in predicting recurrent thrombosis or prognosis; to assess the incidence and severity of PTS; to assess patient-reported QoL; and to assess healthcare resource use for the treatment of cancer-associated VTE.

## Methods

### Study design

**Figure 1:**
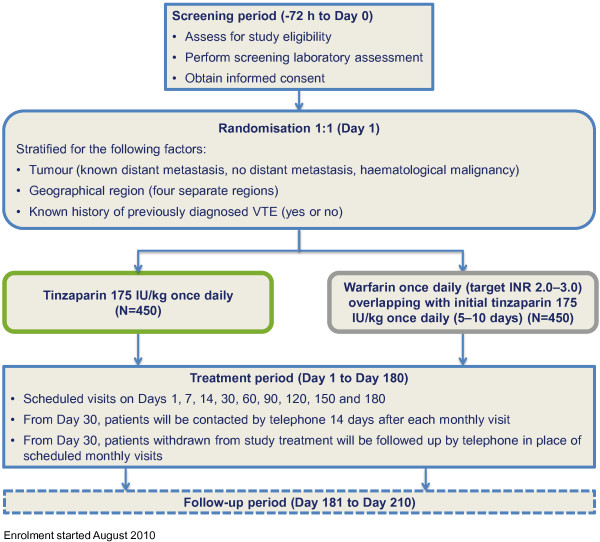
Study design, interventions and timelines.

CATCH is a Phase III, multinational, concealed, randomised, active-controlled, open-label trial with blinded adjudication assessing the efficacy and safety of long-term (6 months) tinzaparin therapy versus anticoagulation with warfarin for the treatment of VTE in cancer patients (Figure 1). It is being conducted in more than 160 sites across five continents.

### Study population

Adult patients (≥18 years or above legal age of consent) with a diagnosis of active cancer and a histologically or cytologically confirmed solid tumour (evidence of early-stage, regionally advanced or metastatic disease) or haematological malignancy are eligible for inclusion. Active cancer is defined by any of the following: diagnosis of cancer within the past 6 months; recurrent, regionally advanced or metastatic disease; any treatment for cancer during the previous 6 months; not in complete remission of a haematological malignancy. Participants must also have symptomatic and objectively confirmed acute proximal DVT and/or PE (Table 2). Other inclusion criteria include Eastern Cooperative Oncology Group (ECOG) performance status of 0, 1, or 2 prior to VTE.

**Table 2:** Diagnostic criteria for VTE

	**VTE before randomisation**	**Recurrent VTE after randomisation**
Symptomatic VTE	• All patients must have diagnostic imaging performed of both legs and the lungs in order to determine baseline presence or absence of DVT or PE.	• Standard objective imaging is required to diagnose recurrent VTE. If there are symptoms from the leg(s) AND lungs, objective imaging is required for both sites.
	• Diagnostic imaging results for DVT:	• Diagnostic imaging results for recurrent DVT:
	- A non-compressible venous segment of the proximal deep veins in the legs, including iliac, femoral and popliteal veins.	- A non-compressible venous segment of the deep veins (proximal and/or distal) in the legs that had normal compression at baseline.
	- An intraluminal filling defect on venography, CT scan or MR venography of the proximal deep veins in the leg.	- A new or extension of 5 cm or greater of intraluminal filling defect on venography, CT scan or MR venography of the deep veins in the leg, including inferior vena cava.
	• Diagnostic imaging results for PE:	- An extension of non-visualisation of the deep veins of the leg in the presence of a sudden cut-off on venography, CT scan or MR venography.
	- An intraluminal filling defect on CT pulmonary angiography.	• Diagnostic imaging results for recurrent PE:
	- A perfusion defect of at least 75% of a segment with a local normal ventilation result (mismatch defect) on ventilation-perfusion lung scintigraphy (high-probability scan).	- A new or extension of an existing intraluminal filling defect on CT pulmonary angiography.
	- A non-high, non-diagnostic ventilation-perfusion lung scan with confirmed DVT.	- A new sudden cut-off of vessels more than 2.5 mm in diameter on CT pulmonary angiography.
		- A new perfusion defect of at least 75% of a segment with a local normal ventilation result (mismatch defect) on ventilation-perfusion lung scintigraphy (high-probability scan).
		- A non-high, non-diagnostic ventilation-perfusion lung scan with confirmed DVT.
		• Diagnostic criteria for fatal PE:
		- Objective testing as above associated with death.
		- Autopsy finding of PE contributing to death.
		- Sudden and unexplained death within the 6-month study period which cannot be attributed to a documented cause and for which PE is the most probable cause.
Incidental VTE	• Not valid as an inclusion criterion.	• Incidental PE or DVT are defined as thrombi that were reported during imaging testing performed for reasons other than for suspected PE or DVT.
	• Diagnosis of incidental VTE during the required baseline imaging represents the baseline status.	• The same diagnostic imaging criteria for recurrent DVT or PE apply to confirming the presence of an incidental DVT or PE.
		• Incidental DVT is only included as an outcome if located in the popliteal or more proximal leg veins.
		• Incidental PE is only included as an outcome if located in segmental or more proximal pulmonary arteries.
		• In patients with incidental PE involving subsegmental pulmonary arteries only, a compression ultrasound showing a new DVT is necessary to confirm a recurrent thrombotic event.

Exclusion criteria include the following: life expectancy <6 months; basal cell carcinoma or non-melanoma skin cancer (in the absence of any other cancer diagnosis); creatinine clearance ≤20 mL/min (according to the abbreviated Modification of Diet in Renal Diseases formula); contraindications to anticoagulation; known hypersensitivity to study medications; history of heparin-induced thrombocytopenia (HIT); therapeutic anticoagulation for >72 hours pre-randomisation; receiving therapeutic anticoagulation at the time of VTE; unlikely to comply with protocol; participation in another interventional study; women of childbearing potential or fertile men not using effective contraception.

The eligibility criteria are very similar to those of previous trials to facilitate cross-trial comparison and possible meta-analysis of the results.

### Consent

Written, informed consent was obtained from all patients for participation in the study after a review of the protocol, their responsibilities and their rights. Consent was also obtained for recording of their data and collection and storage of blood samples, as outlined in the protocol, to allow regulatory monitoring, statistical analysis and peer-review presentation and publication of the study results.

### Randomisation and concealment

Randomisation must occur within 72 hours after the qualifying thrombotic event is objectively confirmed, or within 72 hours of starting therapeutic anticoagulant treatment for a suspected thrombotic event that is then objectively confirmed, whichever time point comes first. Prior to randomisation, all patients must have diagnostic imaging performed for both DVT and PE in order to document asymptomatic DVT or PE at baseline. At randomisation, participants are assigned to either tinzaparin at full treatment doses (175 IU/kg once daily) for 6 months (180 days; for this study, 1 month will equal a period of 30 days) in the experimental arm, or initial tinzaparin treatment for 5–10 days overlapping with dose-adjusted warfarin (target international normalisation ratio [INR] 2.0–3.0) for 6 months in the control arm. Treatment assignment is pre-planned according to a computer-generated randomisation schedule in a 1:1 ratio and concealed until individual randomisation using an interactive voice response system (IVRS) by telephone. In order to balance treatment groups, a stratified randomisation scheme has been applied that takes into account the following: tumour (known distant metastasis, no distant metastasis, haematological malignancy); geographical region (four different regions); and known history of previously diagnosed VTE (yes or no).

### Study treatments

All patients are instructed on the subcutaneous administration of pre-filled syringes of tinzaparin. Patients are given labelled kits containing syringes of tinzaparin containing 0.5 mL (10,000 IU), 0.7 mL (14,000 IU) or 0.9 mL (18,000 IU) of tinzaparin, whichever is most appropriate for their weight. The exact dose of tinzaparin administered once daily is based on the dosage table according to patient body weight at 175 IU/kg, rounded to the nearest 1,000 IU (Table 3). Those assigned to receive tinzaparin for 6 months receive a 30-day supply at the initial visit and then at scheduled monthly visits thereafter. Those assigned to tinzaparin and warfarin receive a 10-day supply of tinzaparin and a 30-day supply of warfarin containing 1-mg, 3-mg and 5-mg tablets. More warfarin is dispensed at follow-up visits. Warfarin is taken once daily at a dose adjusted to maintain a therapeutic INR between 2.0 and 3.0. The use of a warfarin dosing nomogram is encouraged. The first injection of tinzaparin in both groups is administered as soon as possible after randomisation. All patients complete a diary to record the date and time of injections and doses of warfarin, if appropriate. Temporary discontinuation of study drugs not exceeding 3 weeks is permitted for thrombocytopenia (platelet count less than 50 × 10^9^/L), a bleeding event, or if the patient must undergo any invasive procedures. If study drug is held or missed for more than three consecutive weeks, then the patient will be considered to have permanently discontinued study drug.

**Table 3:** Dosage guide for tinzaparin

**175 IU/kg body weight subcutaneously once daily**				
**Syringe size**	**Body weight (kg) rounded up or down to nearest kg**	**Units**	**Volume to expel from syringe prior to injection (mL)**	**Injection volume (mL)**
10,000 IU in 0.5 mL	<34	6,000	0.20	0.30
	35–41	7,000	0.15	0.35
	42–46	8,000	0.10	0.40
	47–51	9,000	0.05	0.45
	52–57	10,000	None	0.50
14,000 IU in 0.7 mL	58–63	11,000	0.15	0.55
	64–67	12,000	0.10	0.60
	68–72	13,000	0.05	0.65
	73–77	14,000	None	0.70
18,000 IU in 0.9 mL	78–83	15,000	0.15	0.75
	84–88	16,000	0.10	0.80
	89–93	17,000	0.05	0.85
	94–103	18,000	None	0.90
**2 ×** 10,000 IU in 0.5 mL	104–124	20,000	None	**2 ×** 0.5
**1 ×** 10,000 IU in 0.5 mL **and 1 ×** 14,000 IU in 0.7 mL	125–145	24,000	None	**1 ×** 0.5 **and 1 ×** 0.7
**2 ×** 14,000 IU in 0.7 mL	146–165	28,000	None	**2 ×** 0.7
**1 ×** 14,000 IU in 0.7 mL **and 1 ×** 18,000 IU in 0.9 mL	166–183	32,000	None	**1 ×** 0.7 **and 1 ×** 0.9

### Primary and secondary efficacy outcomes

The primary composite endpoint is time in days to the first occurrence up to Day 180 of any of the following five objectively documented components: symptomatic DVT; symptomatic non-fatal PE; fatal PE; incidental proximal DVT (popliteal vein or higher); incidental proximal PE (segmental arteries or larger). Each of these components will be considered separately as secondary efficacy endpoints. In addition, a secondary composite efficacy endpoint of symptomatic DVT, symptomatic non-fatal PE and fatal PE will be analysed.

Incidental PE or DVT are defined as thrombi that are reported during imaging testing performed for other reasons (e.g. cancer staging) and not for suspected VTE. Patients may or may not have symptoms of PE or DVT at the time of the test. Incidental PE is only included as an outcome if located in segmental or more central pulmonary arteries; incidental DVT is only included as an outcome if it involves the popliteal or more proximal veins in the legs/pelvis. More distal thrombotic events, such as isolated subsegmental PE and calf DVT, found incidentally are not included in the primary composite efficacy outcome because of conflicting data on their diagnostic accuracy, clinical significance and requirement for anticoagulant treatment [15,35,36].

All of these endpoint thrombotic events must be objectively confirmed using standardised imaging techniques and meet pre-specified diagnostic criteria for recurrent DVT and/or PE (Table 2). Only those events confirmed and validated by the Independent Central Adjudication Committee will be included in the primary efficacy analysis.

### Secondary outcomes

Secondary endpoints include: bleeding (major bleeding, clinically relevant non-major bleeding); all-cause mortality; HIT; risk factors for recurrent VTE; PTS; health-related QoL; and healthcare resource utilisation.

Major bleeding will be defined according to International Society on Thrombosis and Haemostasis (ISTH) criteria [37]: bleeding with a fall in haemoglobin of ≥2 g/dL; bleeding requiring a transfusion of ≥2 units of red cells or whole blood; bleeding that occurs in a critical location, i.e. intracranial, intraspinal, intraocular, retroperitoneal, intraarticular or pericardial; or bleeding that causes death. All non-major bleedings that require any medical or surgical intervention will be classified as ‘clinically relevant non-major bleeding’. Bleeding episodes not meeting either of the above criteria will be classified as trivial bleeding. Only those bleeding events that occur within 24 hours after the last dose of study drug and confirmed by central adjudication will be included in the analysis as a secondary outcome. Bleeding events occurring after that period and up to 1 month after the last dose will be considered as adverse events.

All deaths will be adjudicated and cause of death will be classified as due to: PE (confirmed by autopsy or objective imaging or sudden explained death for which PE is the most probable cause); cancer progression; bleeding; or other causes.

The diagnosis of HIT requires both clinical and laboratory/diagnostic confirmation that is consistent with standard practice, including the use of the Warkentin 4 T score [38]. Laboratory analyses to investigate for HIT are performed locally but will be confirmed at the central laboratory. All cases of reported HIT will be sent for central adjudication.

#### Risk factors for recurrent VTE

VTE risk factors (e.g. tumour type, body mass index [BMI], platelets, haemoglobin and leucocytes) will be assessed at baseline and at the time of a VTE event. Coagulation biomarkers (D-dimer, tissue factor, C-reactive protein, Factor VIII and soluble P-selectin) will be assessed at baseline and end-of-treatment visits.

#### Post-thrombotic syndrome

The presence and severity of PTS will be assessed using the Villalta scale [39] at baseline, each monthly visit and the end of the 1-month post-treatment follow-up period. The Villalta scale classifies PTS as mild if the score is 5–9, moderate if the score is 10–14, and severe if the score is ≥15 or a venous ulcer is present.

#### Patient-reported QoL

Patient-reported QoL will be assessed using the EQ-5D questionnaire (available at http://www.euroqol.org) at baseline, each monthly visit, Day 180 and at the post-treatment follow-up visit. The six-item measure generates a descriptive profile and five of these items can be converted into single index value for health status. The five-item descriptive portion addresses five health dimensions (mobility, self-care, usual activities, pain/discomfort, and anxiety/depression), with respondents indicating one of three possible responses for each dimension. The sixth item is a visual analogue scale (0–100) that is used to report overall health status.

#### Healthcare resource utilisation

Healthcare resource utilisation data will be collected for assessments at each monthly visit and the post-treatment follow-up visit. Only healthcare resources leading to major cost drivers will be collected. These will include resource uses for VTE preventive therapy (i.e. drug, etc.), routine laboratory tests, patient INRs, unscheduled clinical visits, diagnostic tests relevant for VTE diagnosis, blood transfusions, the occurrence of major-bleeding-related events that were possibly or probably due to the study drugs, and costs for the management of patients who develop recurrent VTEs (e.g. hospitalisation).

### Study follow-up

The day of randomisation is considered as Day 1, Visit 1. Subsequent visits occur on Day 7, Day 14, Day 30 (Month 1) and then every 30 days (Months 2–6) until Day 180. Patients stopping study treatment prior to Day 180 for any reason except death are followed up by telephone contacts in place of scheduled monthly visits until Day 180.

Each scheduled visit and telephone contact include a standardised assessment of the signs and symptoms of recurrent VTE. All patients are advised to contact the investigator or delegated site staff if the patient experiences signs and symptoms of VTE between scheduled visits or telephone calls. If signs and symptoms assessed by the investigator or delegated staff are consistent with a recurrent VTE, the patient is advised to return to the clinic urgently for an unscheduled visit for objective testing in order to confirm or refute the suspected recurrent VTE. If the recurrent VTE is confirmed, end-of-treatment assessments are performed and the patient is treated according to local practice and investigator’s discretion. If the recurrent VTE is not confirmed, the patient continues study medication and returns for the next scheduled visit as planned. Diagnosis of symptomatic DVT/PE (both at randomisation and recurrence) must be made by appropriate objective imaging using standard diagnostic criteria (Table 2). Any protocol-defined incidental VTE identified after stopping of study drug will be included in the primary outcome analysis.

All patients are followed for the primary composite efficacy endpoint and for all-cause death up to Day 180 from day of randomisation or death (whichever occurs first), regardless of study drug status. Patients are followed for bleeding events until the last dose of study drug, and for 1 month (30 days) following the last dose of study treatment for all other secondary endpoints. Health-related QoL is followed up to at least Day 180, and for an additional 1 month if Day 180 is the last dose of study treatment. Adverse events and serious adverse events (AE/SAEs) are recorded while a patient is receiving study drug. SAEs are recorded up to 1 month after the last dose of study drug. Coding of AE/SAEs use the Medical Dictionary for Regulatory Activities and severity is graded according to the National Cancer Institute Common Terminology Criteria for AEs v4.03. If a patient dies during the 6-month treatment period, all reasonable efforts will be made to ascertain the following information: the cause of death and obtain a copy of the autopsy report (if available); whether the patient experienced any outcome or SAEs between the last visit or telephone contact and the time of death; and when the last dose of study treatment was taken.

### Study monitoring and oversight

CATCH has approval from the institutional review boards or independent ethics committees of all investigational sites. The trial has been registered with ClinicalTrials.gov (NCT01130025) and was designed in accordance with Good Clinical Practice guidelines.

The Independent Adjudication Committee (IAC) is blinded to study medication assignment and comprises thrombosis, oncology and bleeding experts independent of the study and the study sponsors. The blinded IAC is responsible for adjudicating the following: all efficacy endpoints (i.e. DVTs, PEs and causes of death) occurring up to Day 180; bleeding events occurring up to 24 hours after last dose of study treatment; and HIT events occurring up to 1 month after last dose of study treatment. The adjudication results will be the basis for final analysis of recurrent VTE, bleeding, HIT and deaths.

The independent Data Monitoring Committee comprises clinicians and methodologists independent of the study and the study sponsors and is responsible for monitoring the progress of the study and safety of the patients. The Data Monitoring Committee recommended that the trial continue as planned during its last regular review in August 2012.

### Study management

In close collaboration with the contract research organisation to which the study was outsourced, study-specific training is conducted to ensure protocol adherence and data quality. Initial training of Principal Investigators and site staff (sub-investigators, study coordinators and nurses, pharmacists, and radiologists) was conducted at regional investigator meetings. During the recruitment phase of the study, additional investigator meetings are held regionally, as well as on country and site levels. During the continuous clinical and medical monitoring of the study, a series of help tools have been developed for training and communication purposes. The tools include procedure manuals, guidance documents, fliers, posters and a web-based study portal. A study newsletter is distributed on a monthly basis and sites are supported by visits of senior medical affairs representatives from the sponsor. All sites are monitored clinically to ensure data quality and study protocol adherence. Sites are audited to provide quality control reports, and sites also receive sponsor oversight monitor visits. Data quality and consistency are monitored on a monthly basis using a listing-based approach. Patient profiles are also adopted to ensure patient level oversight, including INR monitoring. Overall study and patient safety is assured by the Data Monitoring Committee. The Steering Committee also convenes regularly to monitor study progress.

### Statistical methods

#### Sample size estimation

This trial aims to demonstrate that long-term treatment with tinzaparin will show greater benefits in reducing the risk of recurrent VTE versus warfarin. Reported incidence rates of the primary endpoint vary in the population of cancer patients. For patients receiving VKA treatment, the CLOT trial supports an incidence rate of 15.8% [28], while meta-analyses support an incidence rate of 12.6% [40] or 14.3% [41]. Our sample size is based on a relative risk reduction of 50%, with an estimated event rate of 12.6% in the control group. Using a time-to-event approach with a two-sided significance level of α = 0.05 and an overall power of 90%, a sample size of 847 patients will be required to detect the above difference. Incorporating an expected dropout rate of 5%, it is planned that 900 patients will be randomised: 450 patients in the tinzaparin arm and 450 patients in the control arm. A sample size re-estimation will be conducted on blinded data when approximately 25% of patients have completed the treatment period, died or been lost to follow-up, to assess the appropriateness of the assumptions. The assumed relative risk reduction of 50% will remain unchanged.

#### Statistical analyses

Descriptive statistics of the patient characteristics will be presented for all randomised patients and for patients grouped according to treatment group and by stratum.

All efficacy criteria will be analysed for the full analysis set (FAS; intention to treat). In addition, the primary efficacy criterion will be analysed for the per-protocol (PP) analysis set and used as supportive data. The efficacy analysis period is defined as the period from randomisation up to 180 days after randomisation and at least 24 hours following the last administration of investigational product. All recurrent VTE outcomes confirmed by central adjudication occurring during this period will be eligible for the primary efficacy analysis. Comparison will be based on the time to first recurrent event per patient. To account for competing risks, Gray’s test will be used for comparing cumulative incidence functions. The cumulative incidence function will be estimated separately for the two treatment groups using a bivariate approach and the corresponding 95% confidence interval (CI) will be computed. The competing risks considered in this study are deaths other than fatal PEs. An interim analysis of the primary endpoint will be performed by the Data Monitoring Committee when 50% of patients have completed treatment, died or been lost to follow-up. The decision will be primarily based on the primary efficacy criterion. The study can be stopped early in the event of superiority or futility.

All secondary efficacy endpoints will be analysed as described for the primary efficacy endpoint. For each time-to-event analysis, the components not defined as the event of interest will be considered a competing risk; 95% CIs will be calculated and *P* values will be corrected for multiplicity using the Hochberg method. For both primary and secondary efficacy analyses, the assessment of recurrent VTE from the blinded IAC will be used; analysis of the investigator assessment of recurrent VTE will not be performed.

Overall mortality will be summarised with Kaplan–Meier estimates and compared between treatment groups using a two-sided log-rank test. An estimate of the treatment effect (hazard ratio and associated CI) will be obtained via Cox regression including all stratification factors. The proportion of patients with a major bleeding event or with clinically relevant non-major bleeding will be compared between groups using Fisher’s exact test. Similar to the primary endpoint, in order to correct for competing risks, a cumulative incidence approach will be used to analyse bleeding data. Competing risks will be deaths from all causes other than fatal bleeding.

An exploratory analysis will be performed to assess the association of clinical baseline characteristics with recurrent symptomatic and/or incidental VTE and bleeding in patients receiving anticoagulant therapy. The presence and severity of PTS will be analysed using the Villalta scale; comparison within and between treatment groups will be performed in addition to analysis of the change in severity over time. For assessment of health-related QoL, descriptive statistics will be produced for the five EQ-5D dimensions (i.e. mobility, self-care, usual activities, pain/discomfort, and anxiety/depression), visual analogue scale, and utility index at each assessment for patients in the tinzaparin and control groups. Dimensional outcomes and utility indices of the EQ-5D will then be assessed using multivariate mixed models for a repeated-measures analysis. As a secondary endpoint in the QoL analysis, ‘clinical stability’ will be evaluated, defined as an improvement or <10% drop in the utility index of the EQ-5D when measured during two consecutive monthly assessments. Clinical stability and deterioration between treatment groups will be analysed using logistic regression analysis, Kaplan–Meier curves and Cox proportional hazards regression. Healthcare resource utilisation associated with the prevention of secondary VTEs will be assessed between groups for a reference country using non-parametric statistical techniques such as quantile regression analysis. In addition, the incremental cost per VTE avoided and quality-adjusted life year (QALY) gained with tinzaparin will also be determined.

## Results and discussion

The main aim of the CATCH trial is to confirm the superior efficacy of tinzaparin over warfarin for the secondary prevention of VTE in patients with active cancer. CATCH will extend and broaden the findings from previous clinical trials comparing LMWH with a VKA such as warfarin by addressing several current knowledge gaps in the management of VTE in patients with cancer. These include the generation of prospective data on the clinical significance of incidental VTE and the potential identification of risk factors predicting recurrence, both of which will add information that may help to further tailor therapy. CATCH will also include specific assessment of the incidence and severity of PTS in patients with cancer. This has not previously been performed despite PTS being a common and burdensome complication of DVT [42]; the results will add to existing data that have shown that long-term treatment with tinzaparin significantly reduces the incidence of PTS and leg ulcers in a broader patient population with acute DVT [43]. Little information exists concerning the needs and preferences of cancer patients at risk of recurrent VTE [24]; the patient-reported outcomes from CATCH will provide important insights into QoL aspects of patient care. Finally, unlike the CLOT trial, which used a reduced dose of dalteparin after 1 month, CATCH will use tinzaparin at the full therapeutic dose for 6 months. The rationale for choosing this dose and several key design features of the CATCH trial are discussed below.

### Rationale for tinzaparin and therapeutic dose in the trial

There are pharmacological and clinical data to support the use of tinzaparin at a therapeutic dose for the treatment of cancer-associated thrombosis. Tinzaparin has the highest mean molecular weight (approximately 6,500 Da) of the commercially available LMWHs and is least dependent on renal clearance [44]. This is supported by findings from clinical studies, which have shown that treatment doses of tinzaparin do not accumulate in patients with mild to severe renal insufficiency (clearance ≥20 mL/min) [45,46]. Tinzaparin can be used in elderly patient populations without any dose reductions at both prophylactic (4500 anti-Xa IU) and therapeutic doses (175 anti-Xa IU/kg) [45-47]. The elimination profile of tinzaparin is an important consideration among patients with cancer, of whom many are elderly or have renal impairment. Tinzaparin is also the most reversible of any LMWH, with 65–85% of its anti-Xa activity neutralised by protamine sulphate [48-50].

Prolonged treatment with tinzaparin at a therapeutic dose (175 anti-Xa IU/kg) to prevent VTE recurrence has been shown to be effective and safe in several clinical studies [27,43,51-53]. In particular, the effect of tinzaparin (175 anti-Xa IU/kg) was evaluated over a 90-day period in the Main-LITE study of acute symptomatic DVT, in which approximately one quarter of subjects had cancer [27,52]. In the 200 patients with cancer, the primary outcome of VTE recurrence at the end of the 3-month treatment period was 6% in those receiving tinzaparin and 10% in those receiving VKA (difference -4.0; 95% CI -12.0 to 4.1); at 12 months, the respective rates were 7% versus 16% (*P* = 0.04). Treatment with tinzaparin was not associated with an increased risk of bleeding compared with the VKA arm. In addition, a related study (Home-LITE) compared long-term treatment at home with tinzaparin or usual care in a broad population of patients with DVT and showed that tinzaparin use was associated with a lower risk of PTS [43]. The single, therapeutic-dose regimen of tinzaparin in CATCH will simplify treatment and perhaps produce lower risks of recurrent VTE.

### Rationale for warfarin as comparator in the control group

Initial consideration was given to using dalteparin as the control group comparator because it is the only LMWH approved for extended use in the prevention of recurrent VTE in patients with cancer, but it is still not considered the standard of care in many countries and warfarin remains a commonly used anticoagulant in cancer patients worldwide [54-58]. This may be a reflection of the undesirable mode of drug delivery of LMWHs (requiring once- or twice-daily subcutaneous injections), as well as their substantially higher drug cost compared with VKAs.

Furthermore, comparing tinzaparin with dalteparin with efficacy as the primary outcome would require a non-inferiority study design, as there is no evidence that one LMWH is superior in efficacy over another. In fact, a randomised controlled trial that compared tinzaparin with dalteparin for the initial treatment of VTE in a mixed population of non-cancer and cancer patients found no difference between the two LMWHs [59]. Based on a planned interim analysis, the investigators determined that a study including 16,433 per group would be needed to demonstrate a statistically significant difference between the two LMWHs. Therefore, the study was terminated early based on this futility rationale. To perform a non-inferiority study to compare tinzaparin with dalteparin would also seem futile in advancing patient care, as the two drugs are already commonly used interchangeably and both are recommended for extended treatment of cancer-associated thrombosis.

Consequently, we believe that it is more beneficial to patient care and scientific advancement if a robust, well-designed randomised trial is conducted to compare tinzaparin with warfarin and to confirm the results reported in the previous trials. Such a Phase III trial also provides the opportunity to prospectively investigate important clinical outcomes that have not been addressed and which are relevant for patients and support individualised care, including PTS, patient-reported QoL, predictors and markers of recurrent VTE and bleeding, and healthcare resource utilisation.

### Rationale for open-label design

An open-label design could be a source of potential bias. However, a double-blind study in oncology patients would be ethically and practically challenging as it would expose them to excessive burden: subcutaneous injections of a placebo drug in patients assigned to warfarin treatment and placebo tablets with sham INR monitoring requiring multiple unnecessary blood draws in patients assigned to long-term tinzaparin treatment. It is also very difficult to generate convincing or realistic sham INRs in those patients receiving long-term tinzaparin to reflect changes that often result from chemotherapy. Furthermore, it is potentially dangerous to mask the anticoagulant used in those with severe thrombocytopenia. For example, a false positive result on a HIT assay in a patient who is receiving warfarin therapy could be switched to argatroban for HIT treatment. Lastly, masking study drug frequently heightens patient anxiety and this is a particularly sensitive issue in oncology patients in whom the risks of bleeding and recurrent VTE are so much higher than in the general population. Hence, an open-label design was chosen for patient safety and preference and to minimise the negative impact on their QoL. To minimise the potential bias of this design, all reported VTE and bleeding events, HIT and cause of death are centrally adjudicated by an independent expert committee that is blinded to treatment assignment.

### Rationale for including incidental VTE as an efficacy endpoint

Incidental VTE is now commonly reported in cancer patients because of the prevalence of routine CT imaging for follow-up. Although the exact history and optimal treatment of incidental thrombotic events remain uncertain, we included these events in the primary composite endpoint for several reasons: 1) the majority of these events are associated with symptoms consistent with PE or DVT [14]; 2) several studies have shown that incidental VTEs are clinically significant events with similar prognosis to symptomatic VTE [60,61]; 3) expert consensus guidelines recommend treatment with therapeutic anticoagulation when incidental proximal DVT or PE are reported [15]. In addition, emerging evidence in cancer patients shows that asymptomatic VTE rates correlate directly with symptomatic VTE rates and survival [16,17].

## Conclusions

The results obtained from the CATCH trial will add significantly to the knowledge of the efficacy, safety and cost effectiveness of the LMWH tinzaparin in the prevention of recurrent VTE in cancer patients. The findings should provide greater therapeutic choice and better understanding of the long-term outcomes of patients with cancer and thrombosis.

## Competing interests

AYYL has received payment for honoraria (LEO Pharma, Pfizer, Sanofi Aventis, Bayer, Boehringer Ingelheim) and research funding (LEO Pharma, Eisai). RB declares no competing interests. MSJ and MFJ are employees of LEO Pharma. PWK has received payment for research funding (LEO Pharma). GM has received payment for research funding (LEO Pharma, Boehringer Ingelheim). AAK has received payment for honoraria (LEO Pharma).

## Authors’ contributions

AYYL is the international coordinating investigator of the study. All authors serve on the Steering Committee and have input in the study design and oversight of the conduct and progress of the study. The manuscript has been prepared, reviewed and approved by all members of the Steering Committee.

## Pre-publication history

The pre-publication history for this paper can be accessed here:

http://www.biomedcentral.com/1471-2407/13/284/prepub

## References

[B1] Blom JW Doggen CJ Osanto S Rosendaal FR Malignancies, prothrombotic mutations, and the risk of venous thrombosis JAMA 2005 293 715 722 10.1001/jama.293.6.715 15701913

[B2] Blom JW Vanderschoot JP Oostindiër MJ Osanto S van der Meer FJ Rosendaal FR Incidence of venous thrombosis in a large cohort of 66,329 cancer patients: results of a record linkage study J Thromb Haemost 2006 4 529 535 10.1111/j.1538-7836.2006.01804.x 16460435

[B3] Levitan N Dowlati A Remick SC Tahsildar HI Sivinski LD Beyth R Rimm AA Rates of initial and recurrent thromboembolic disease among patients with malignancy versus those without malignancy. Risk analysis using Medicare claims data Medicine (Baltimore) 1999 78 285 291 10.1097/00005792-199909000-00001 10499070

[B4] Khorana AA Francis CW Culakova E Kuderer NM Lyman GH Thromboembolism is a leading cause of death in cancer patients receiving outpatient chemotherapy J Thromb Haemost 2007 5 632 634 10.1111/j.1538-7836.2007.02374.x 17319909

[B5] Sørensen HT Mellemkjær L Olsen JH Baron JA Prognosis of cancers associated with venous thromboembolism N Engl J Med 2000 343 1846 1850 10.1056/NEJM200012213432504 11117976

[B6] Khorana AA Francis CW Culakova E Lyman GH Risk factors for chemotherapy-associated venous thromboembolism in a prospective observational study Cancer 2005 104 2822 2829 10.1002/cncr.21496 16284987

[B7] Khorana AA Connolly GC Assessing risk of venous thromboembolism in the patient with cancer J Clin Oncol 2009 27 4839 4847 10.1200/JCO.2009.22.3271 19720906 PMC2764392

[B8] Sallah S Wan JY Nguyen NP Venous thrombosis in patients with solid tumors: determination of frequency and characteristics Thromb Haemost 2002 87 575 579 12008937

[B9] Falanga A The incidence and risk of venous thromboembolism associated with cancer and nonsurgical cancer treatment Cancer Invest 2009 27 105 115 10.1080/07357900802563028 19160098

[B10] Jacobson G Lammli J Zamba G Hua L Goodheart MJ Thromboembolic events in patients with cervical carcinoma: incidence and effect on survival Gynecol Oncol 2009 113 240 244 10.1016/j.ygyno.2009.01.021 19251310

[B11] Nalluri SR Chu D Keresztes R Zhu X Wu S Risk of venous thromboembolism with the angiogenesis inhibitor bevacizumab in cancer patients: a meta-analysis JAMA 2008 300 2277 2285 10.1001/jama.2008.656 19017914

[B12] Starling N Rao S Cunningham D Iveson T Nicolson M Coxon F Middleton G Daniel F Oates J Norman AR Thromboembolism in patients with advanced gastroesophageal cancer treated with anthracycline, platinum, and fluoropyrimidine combination chemotherapy: a report from the UK National Cancer Research Institute Upper Gastrointestinal Clinical Studies Group J Clin Oncol 2009 27 3786 3793 10.1200/JCO.2008.19.4274 19398575

[B13] Maraveyas A Johnson M Does clinical method mask significant VTE-related mortality and morbidity in malignant disease? Br J Cancer 2009 100 1837 1841 10.1038/sj.bjc.6605091 19491905 PMC2714244

[B14] O'Connell CL Boswell WD Duddalwar V Caton A Mark LS Vigen C Liebman HA Unsuspected pulmonary emboli in cancer patients: clinical correlates and relevance J Clin Oncol 2006 24 4928 4932 10.1200/JCO.2006.06.5870 17050877

[B15] Kearon C Akl EA Comerota AJ Prandoni P Bounameaux H Goldhaber SZ Nelson ME Wells PS Gould MK Dentali F Crowther M Kahn SR Antithrombotic therapy for VTE disease: antithrombotic therapy and prevention of thrombosis, 9th ed: American College of Chest Physicians evidence-based clinical practice guidelines Chest 2012 141 Suppl 2 e419S e494S 22315268 10.1378/chest.11-2301 PMC3278049

[B16] Dentali F Ageno W Becattini C Galli L Gianni M Riva N Imberti D Squizzato A Venco A Agnelli G Prevalence and clinical history of incidental, asymptomatic pulmonary embolism: a meta-analysis Thromb Res 2010 125 518 522 10.1016/j.thromres.2010.03.016 20451960

[B17] Menapace LA Peterson DR Berry A Sousou T Khorana AA Symptomatic and incidental thromboembolism are both associated with mortality in pancreatic cancer Thromb Haemost 2011 106 371 378 10.1160/TH10-12-0789 21713322

[B18] Khorana AA Cancer and thrombosis: implications of published guidelines for clinical practice Ann Oncol 2009 20 1619 1630 10.1093/annonc/mdp068 19561038

[B19] Petersen LJ Anticoagulation therapy for prevention and treatment of venous thromboembolic events in cancer patients: a review of current guidelines Cancer Treat Rev 2009 35 754 764 10.1016/j.ctrv.2009.08.009 19762155

[B20] Hutten BA Prins MH Gent M Ginsberg J Tijssen JG Büller HR Incidence of recurrent thromboembolic and bleeding complications among patients with venous thromboembolism in relation to both malignancy and achieved international normalized ratio: a retrospective analysis J Clin Oncol 2000 18 3078 3083 10963635 10.1200/JCO.2000.18.17.3078

[B21] Prandoni P Lensing AW Piccioli A Bernardi E Simioni P Girolami B Marchiori A Sabbion P Prins MH Noventa F Girolami A Recurrent venous thromboembolism and bleeding complications during anticoagulant treatment in patients with cancer and venous thrombosis Blood 2002 100 3484 3488 10.1182/blood-2002-01-0108 12393647

[B22] Carrier M Le Gal G Cho R Tierney S Rodger M Lee AY Dose escalation of low molecular weight heparin to manage recurrent venous thromboembolic events despite systemic anticoagulation in cancer patients J Thromb Haemost 2009 7 760 765 10.1111/j.1538-7836.2009.03326.x 19245418

[B23] Lee AY Anticoagulation in the treatment of established venous thromboembolism in patients with cancer J Clin Oncol 2009 27 4895 4901 10.1200/JCO.2009.22.3958 19738121

[B24] Noble SI Finlay IG Is long-term low-molecular-weight heparin acceptable to palliative care patients in the treatment of cancer related venous thromboembolism? A qualitative study Palliat Med 2005 19 197 201 10.1191/0269216305pm1008oa 15920933

[B25] Weitz JI Low-molecular-weight heparins N Engl J Med 1997 337 688 698 10.1056/NEJM199709043371007 9278467

[B26] Deitcher SR Kessler CM Merli G Rigas JR Lyons RM Fareed J Secondary prevention of venous thromboembolic events in patients with active cancer: enoxaparin alone versus initial enoxaparin followed by warfarin for a 180-day period Clin Appl Thromb Hemost 2006 12 389 396 10.1177/1076029606293692 17000884

[B27] Hull RD Pineo GF Brant RF Mah AF Burke N Dear R Wong T Cook R Solymoss S Poon MC Raskob G for the LITE Trial Investigators Long-term low-molecular-weight heparin versus usual care in proximal-vein thrombosis patients with cancer Am J Med 2006 119 1062 1072 10.1016/j.amjmed.2006.02.022 17145251

[B28] Lee AY Levine MN Baker RI Bowden C Kakkar AK Prins M Rickles FR Julian JA Haley S Kovacs MJ Gent M Low-molecular-weight heparin versus a coumarin for the prevention of recurrent venous thromboembolism in patients with cancer N Engl J Med 2003 349 146 153 10.1056/NEJMoa025313 12853587

[B29] Meyer G Marjanovic Z Valcke J Lorcerie B Gruel Y Solal-Celigny P Le Maignan C Extra JM Cottu P Farge D Comparison of low-molecular-weight heparin and warfarin for the secondary prevention of venous thromboembolism in patients with cancer: a randomized controlled study Arch Intern Med 2002 162 1729 1735 10.1001/archinte.162.15.1729 12153376

[B30] Lyman GH Khorana AA Falanga A Clarke-Pearson D Flowers C Jahanzeb M Kakkar A Kuderer NM Levine MN Liebman H Mendelson D Raskob G Somerfield MR Thodiyil P Trent D Francis CW American Society of Clinical Oncology guideline: recommendations for venous thromboembolism prophylaxis and treatment in patients with cancer J Clin Oncol 2007 25 5490 5505 10.1200/JCO.2007.14.1283 17968019

[B31] Mandalà M Falanga A Roila F Management of venous thromboembolism in cancer patients: ESMO clinical recommendations Ann Oncol 2009 20 Suppl 4 iv182 iv184 10.1093/annonc/mdp167 19454449

[B32] National Comprehensive Cancer Network NCCN Clinical Practice Guidelines in Oncology (NCCN Guidelines^®^). Venous Thromboembolic Disease 2013 Version 2.2013. Accessible via http://www.nccn.org/clinical.asp.

[B33] Cherkowski G Dietrich J Chen F Fryzek J Bridges K Heparin use and venous thromboembolism (VTE) among cancer patients receiving chemotherapy with a prior history of VTE J Clin Oncol 2009 27 15s abst 6616

[B34] Simons WR Choe Y Powers A McQueen C A real-world evaluation of the effectiveness of dalteparin in the prevention of recurrent venous thromboembolism compared to warfarin in patients with cancer J Clin Oncol 2010 28 15s abst 9115

[B35] Goodman LR Small pulmonary emboli: what do we know? Radiology 2005 234 654 658 10.1148/radiol.2343041326 15734923

[B36] O'Connell C Razavi P Ghalichi M Boyle S Vasan S Mark L Caton A Duddalwar V Boswell W Grabow K Liebman HA Unsuspected pulmonary emboli adversely impact survival in patients with cancer undergoing routine staging multi-row detector computed tomography scanning J Thromb Haemost 2011 9 305 311 10.1111/j.1538-7836.2010.04114.x 20955348

[B37] Schulman S Kearon C Definition of major bleeding in clinical investigations of antihemostatic medicinal products in non-surgical patients J Thromb Haemost 2005 3 692 694 10.1111/j.1538-7836.2005.01204.x 15842354

[B38] Linkins L-A Dans AL Moores LK Bona R Davidson BL Schulman S Crowther M Treatment and prevention of heparin-induced thrombocytopenia: antithrombotic therapy and prevention of thrombosis, 9th ed: American College of Chest Physicians evidence-based clinical practice guidelines Chest 2012 141 e495S e530S 10.1378/chest.11-2303 22315270 PMC3278058

[B39] Kahn SR Post-thrombotic syndrome after deep venous thrombosis: risk factors, prevention, and therapeutic options Clin Adv Hematol Oncol 2009 7 433 435 19701149

[B40] Ferretti G Bria E Giannarelli D Carlini P Felici A Mandalà M Papaldo P Fabi A Ciccarese M Cuppone F Cecere FL Nuzzo C Terzoli E Cognetti F Is recurrent venous thromboembolism after therapy reduced by low-molecular-weight heparin compared with oral anticoagulants? Chest 2006 130 1808 1816 10.1378/chest.130.6.1808 17167001

[B41] Akl EA Labedi N Barba M Terrenato I Sperati F Muti P Schunemann H Anticoagulation for the long-term treatment of venous thromboembolism in patients with cancer Cochrane Database Syst Rev 2011 6 CD006650 21678361 10.1002/14651858.CD006650.pub3

[B42] Hull RD Liang J Townshend G Long-term low-molecular-weight heparin and the post-thrombotic syndrome: a systematic review Am J Med 2011 124 756 765 10.1016/j.amjmed.2011.02.033 21787905

[B43] Hull RD Pineo GF Brant R Liang J Cook R Solymoss S Poon MC Raskob G for the LITE Trial Investigators Home therapy of venous thrombosis with long-term LMWH versus usual care: patient satisfaction and post-thrombotic syndrome Am J Med 2009 122 762 769 10.1016/j.amjmed.2008.12.023 19635277

[B44] Hoy SM Scott LJ Plosker GL Tinzaparin sodium: a review of its use in the prevention and treatment of deep vein thrombosis and pulmonary embolism, and in the prevention of clotting in the extracorporeal circuit during haemodialysis Drugs 2010 70 1319 1347 10.2165/11203710-000000000-00000 20568836

[B45] Pautas E Gouin I Bellot O Andreux JP Siguret V Safety profile of tinzaparin administered once daily at a standard curative dose in two hundred very elderly patients Drug Saf 2002 25 725 733 10.2165/00002018-200225100-00005 12167068

[B46] Siguret V Pautas E Février M Wipff C Durand-Gasselin B Laurent M Andreux JP d'Urso M Gaussem P Elderly patients treated with tinzaparin (Innohep^®^) administered once daily (175 anti-Xa IU/kg): anti-Xa and anti-IIa activities over 10 days Thromb Haemost 2000 84 800 804 11127859

[B47] Mahé I Aghassarian M Drouet L Bal Dit-Sollier C Lacut K Heilmann JJ Mottier D Bergmann JF Tinzaparin and enoxaparin given at prophylactic dose for eight days in medical elderly patients with impaired renal function: a comparative pharmacokinetic study Thromb Haemost 2007 97 581 586 17393021

[B48] Holst J Lindblad B Bergqvist D Garre K Nielsen H Hedner U Østergaard PB Protamine neutralization of intravenous and subcutaneous low-molecular-weight heparin (tinzaparin, Logiparin). An experimental investigation in healthy volunteers Blood Coagul Fibrinolysis 1994 5 795 803 10.1097/00001721-199410000-00018 7865687

[B49] Schroeder M Hogwood J Gray E Mulloy B Hackett A-M Johansen KB Protamine neutralisation of low molecular weight heparins and their oligosaccharide components Anal Bioanal Chem 2011 399 763 771 10.1007/s00216-010-4220-8 20922518

[B50] Crowther MA Berry LR Monagle PT Chan AK Mechanisms responsible for the failure of protamine to inactivate low-molecular-weight heparin Br J Haematol 2002 116 178 186 10.1046/j.1365-2141.2002.03233.x 11841415

[B51] Daskalopoulos ME Daskalopoulou SS Tzortzis E Sfiridis P Nikolaou A Dimitroulis D Kakissis I Liapis CD Long-term treatment of deep venous thrombosis with a low molecular weight heparin (tinzaparin): a prospective randomized trial Eur J Vasc Endovasc Surg 2005 29 638 650 10.1016/j.ejvs.2004.02.029 15878544

[B52] Hull RD Pineo GF Brant RF Mah AF Burke N Dear R Wong T Cook R Solymoss S Poon MC Raskob G for the LITE Trial Investigators Self-managed long-term low-molecular-weight heparin therapy: the balance of benefits and harms Am J Med 2007 120 72 82 10.1016/j.amjmed.2006.03.030 17208082

[B53] Romera A Cairols MA Vila-Coll R Martí X Colomé E Bonell A Lapiedra O A randomised open-label trial comparing long-term sub-cutaneous low-molecular-weight heparin compared with oral-anticoagulant therapy in the treatment of deep venous thrombosis Eur J Vasc Endovasc Surg 2009 37 349 356 10.1016/j.ejvs.2008.11.030 19121589

[B54] Bounameaux H Spirk D Kucher N A nation-wide initiative against venous thromboembolism Swiss Med Wkly 2011 141 w13241 21720971 10.4414/smw.2011.13241

[B55] Delate T Witt DM Ritzwoller D Weeks JC Kushi L Hornbrook MC Aiello Bowles EJ Schrag D Outpatient use of low molecular weight heparin monotherapy for first-line treatment of venous thromboembolism in advanced cancer Oncologist 2012 17 419 427 10.1634/theoncologist.2011-0323 22334451 PMC3316928

[B56] Trujillo-Santos J Nieto JA Tiberio G Piccioli A Di Micco P Prandoni P Monreal M Predicting recurrences or major bleeding in cancer patients with venous thromboembolism. Findings from the RIETE Registry Thromb Haemost 2008 100 435 439 18766259

[B57] Spirk D Ugi J Korte W Husmann M Hayoz D Baldi T Frauchiger B Banyai M Aujesky D Baumgartner I Kucher N Long-term anticoagulation treatment for acute venous thromboembolism in patients with and without cancer. The SWIss Venous ThromboEmbolism Registry (SWIVTER) II Thromb Haemost 2011 105 962 967 10.1160/TH11-01-0002 21475778

[B58] Imberti D Agnelli G Ageno W Moia M Palareti G Pistelli R Rossi R Verso M Clinical characteristics and management of cancer-associated acute venous thromboembolism: findings from the MASTER Registry Haematologica 2008 93 273 278 10.3324/haematol.11458 18223291

[B59] Wells PS Anderson DR Rodger MA Forgie MA Florack P Touchie D Morrow B Gray L O'Rourke K Wells G Kovacs J Kovacs MJ A randomized trial comparing 2 low-molecular-weight heparins for the outpatient treatment of deep vein thrombosis and pulmonary embolism Arch Intern Med 2005 165 733 738 10.1001/archinte.165.7.733 15824291

[B60] Piatek C O'Connell C Unsuspected pulmonary embolism: impact on mortality among cancer patients Curr Opin Pulm Med 2012 18 406 409 10.1097/MCP.0b013e328355392a 22759768

[B61] den Exter PL Jiménez D Kroft LJ Huisman MV Outcome of incidentally diagnosed pulmonary embolism in patients with malignancy Curr Opin Pulm Med 2012 18 399 405 10.1097/MCP.0b013e3283553914 22634737

